# Development and evaluation of a diagnostic cytokine-release assay for *Mycobacterium suricattae* infection in meerkats (*Suricata suricatta*)

**DOI:** 10.1186/s12917-016-0927-x

**Published:** 2017-01-04

**Authors:** Charlene Clarke, Stuart James Patterson, Julian Ashley Drewe, Paul David van Helden, Michele Ann Miller, Sven David Charles Parsons

**Affiliations:** 1 SAMRC Centre for TB Research; DST/NRF Centre of Excellence for Biomedical Tuberculosis Research; Division of Molecular Biology and Human Genetics, Faculty of Medicine and Health Sciences, Stellenbosch University, Cape Town, South Africa; 2 Veterinary Epidemiology, Economics and Public Health Group, Royal Veterinary College, Hawkshead Lane, North Mymms, Hertfordshire, AL9 7TA UK

**Keywords:** Cytokine, Diagnosis, IP-10, Meerkat, *Mycobacterium suricattae*, Tuberculosis

## Abstract

**Background:**

Sensitive diagnostic tools are necessary for the detection of *Mycobacterium suricattae* infection in meerkats (*Suricata suricatta*) in order to more clearly understand the epidemiology of tuberculosis and the ecological consequences of the disease in this species. We therefore aimed to develop a cytokine release assay to measure antigen-specific cell-mediated immune responses of meerkats.

**Results:**

Enzyme-linked immunosorbent assays (ELISAs) were evaluated for the detection of interferon-gamma (IFN-γ) and IFN-γ inducible protein 10 (IP-10) in meerkat plasma. An IP-10 ELISA was selected to measure the release of this cytokine in whole blood in response to Bovigam® PC-HP Stimulating Antigen, a commercial peptide pool of *M. bovis* antigens. Using this protocol, captive meerkats with no known *M. suricattae* exposure (*n* = 10) were tested and results were used to define a diagnostic cut off value (mean plus 2 standard deviations). This IP-10 release assay (IPRA) was then evaluated in free-living meerkats with known *M. suricattae* exposure, categorized as having either a low, moderate or high risk of infection with this pathogen. In each category, respectively, 24.7%, 27.3% and 82.4% of animals tested IPRA-positive. The odds of an animal testing positive was 14.0 times greater for animals with a high risk of *M. suricattae* infection compared to animals with a low risk.

**Conclusion:**

These results support the use of this assay as a measure of *M. suricattae* exposure in meerkat populations. Ongoing longitudinal studies aim to evaluate the value of the IPRA as a diagnostic test of *M. suricattae* infection in individual animals.

**Electronic supplementary material:**

The online version of this article (doi:10.1186/s12917-016-0927-x) contains supplementary material, which is available to authorized users.

## Background

**Fig. 1:**
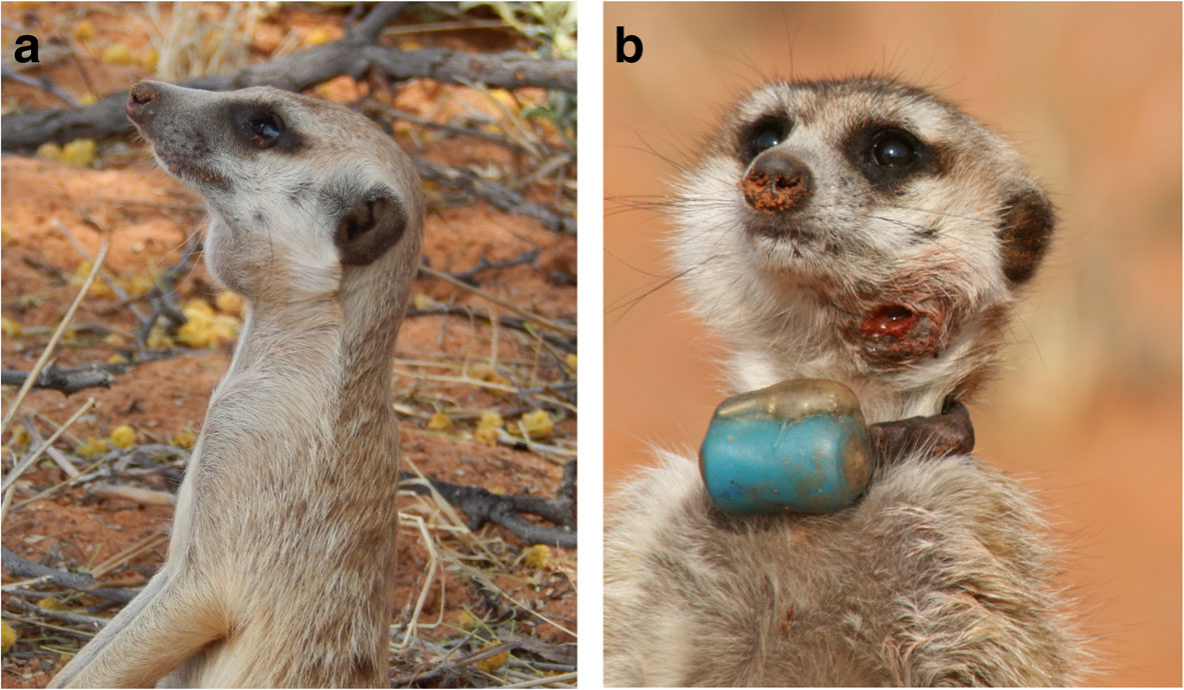
Meerkats with pathology typical of tuberculosis caused by *Mycobacterium suricattae*. **a** A meerkat displaying swelling of the submandibular lymph node. These lesions typically present at post mortem as granulomatous hyperplasia. **b** A meerkat with a draining sinus tract following necrosis and abscessation of the submandibular lymph node

In the Kalahari Desert of South Africa, tuberculosis (TB) caused by *Mycobacterium suricattae* results in morbidity and mortality in meerkats [1, 2]. In this species, TB presents as disseminated disease primarily affecting the spleen, liver, lungs and head lymph nodes and a characteristic clinical finding is swelling of the sub-mandibular lymph nodes [1] (Fig. 1). Clinical disease often progresses to mortality resulting in changes in population ecology through group-level extinctions [3].

Improved diagnostic tests for TB in meerkats are necessary to advance the understanding of disease epidemiology and may allow the development of an effective TB control strategy. Serological assays for TB have previously been shown to have low sensitivity in this species [4]; however, assays detecting cell-mediated immune responses have, to date, not been evaluated [5]. In other species, such as cattle, in vitro tests of cell-mediated immunity (CMI) offer the most sensitive methods for TB diagnosis by detecting the release of interferon-gamma (IFN-γ) in response to mycobacterial antigens [6, 7]. Moreover, an alternative diagnostic marker of CMI, IFN-γ-inducible protein 10 (IP-10), has been shown to improve diagnostic sensitivity in humans and buffaloes (*Syncerus caffer*) [8, 9]. The specificity of such assays can be optimised by using highly specific antigens such as 6 kDa early secretory antigenic target (ESAT-6) and 10 kDa culture filtrate protein (CFP-10) [10, 11]. However, the genes encoding these proteins have been deleted from *M. suricattae* and they are therefore unlikely to be suitable diagnostic antigens for this pathogen [2]. As an alternative, the commercially available Bovigam® PC-HP peptide pool, which contains ESAT-6 and CFP-10 peptides, includes antigens derived from the gene *Rv*3615*c* and an additional 3 genes, and could be useful for the detection of *M. suricattae* infection [11].

The purposes of this study were, therefore, to develop enzyme-linked immunosorbent assays (ELISAs) for the measurement of IFN-γ and IP-10 in meerkat plasma, evaluate the diagnostic utility of the PC-HP peptide pool in this species, and assess the test performance of an optimised diagnostic assay for *M. suricattae* infection in a population of free-living meerkats.

## Materials and methods

### Animals

Captive meerkats with no known history of *M. suricattae* exposure served as an uninfected control group for the development of the cytokine release assay. These animals (*n* = 10) were housed in two groups on a natural soil substrate in an enriched environment in enclosures (6 × 10 meters) at Giraffe House, a wildlife awareness centre situated in Stellenbosch, South Africa. They were fed eggs and day-old chicks or chicken meat daily and had free access to clean water.

Animals with known exposure to *M. suricattae* were opportunistically sampled from a population of free-living meerkats, which have been habituated to the presence of researchers [12], from the Kuruman River Reserve, Northern Cape, South Africa (26°58’S, 21°49’E). Between September 2014 and February 2015, animals from this population were sampled from 12 social groups. Hereafter, animals were classified according to their presumed infection risk: low risk (category 1), comprising animals from social groups with no known history of TB; intermediate risk (category 2), comprising animals from social groups with either one or two known deaths due to TB in the preceding two years; and high risk (category 3), comprising meerkats from social groups with more than two deaths due to TB in the preceding two years.

Permission to perform the study was obtained from the University of Stellenbosch Animal Ethics committee (Reference no. SU-ACUM14-00042). Permits to conduct animal research were obtained from the Northern Cape Department of Environment and Nature Conservation (Permit no. FAUNA 194/2014) and the National Department of Agriculture, Forestry and Fisheries (Reference no. 12/11/1/7/3).

### Blood collection and processing

Meerkats were captured by hand and placed in cotton bags or caught in nets and physically restrained with towels prior to induction and maintenance of anaesthesia with isoflurane (Safeline Pharmaceuticals Ltd, Roodepoort, SA) via facemask. Using a 25G needle and syringe, 2 ml blood was collected from the jugular vein and transferred to a heparinised blood tube (Greiner Bio-one, Kremsmünster, Austria). Animals were monitored after completion of the procedure and returned to their natural environment once fully recovered.

Aliquots of whole blood (150 μl) were transferred to each of three 1.5 ml microcentrifuge tubes containing, respectively, 15 μl phosphate buffered saline (PBS); 15 μl PC-HP peptide solution (Prionics AG, Schlieren, Switzerland), prepared according to the manufacturer’s instructions; and 15 μl pokeweed mitogen (PWM) solution in PBS (final concentration 50 μg/ml). Tubes were thoroughly mixed, incubated at 37 °C for 24 hours and centrifuged at 1300 × g for 6 minutes, after which the plasma was harvested and stored at - 80 °C.

### ELISA protocol

All ELISAs described below were performed according to the following protocol. Capture antibody (Table 1) in PBS (50 μl) was used to coat wells of 96-well MaxiSorp polystyrene ELISA plates (Thermo Fisher Scientific, Massachusetts, USA) which were incubated at 4 °C overnight. All subsequent steps were performed at room temperature. Plates were washed three times with wash buffer (WB) consisting of 0.05% Tween-20 (Sigma-Aldrich, Missouri, USA) in PBS and blocked with 200 μl/well blocking buffer (BB) comprising WB with 0.1% bovine serum albumin (Roche, Basel, Switzerland). After 1 h incubation, plates were washed again. For IFN-ɣ assays, 25 μl of each plasma sample was incubated with 25 μl BB and for IP-10 assays, 12.5 μl of each plasma sample with 37.5 μl BB. After 2 h, plates were washed and incubated for 1 h with 50 μl/well biotinylated detection antibody (Table 1) diluted in BB. After washing, plates were incubated for an additional 1 h with 50 μl/well streptavidin-horseradish peroxidase (R&D systems, Minnesota, USA) diluted 1:200 in BB. After a final wash step, plates were incubated for 20 minutes with 50 μl/well tetramethylbenzidine (TMB) substrate solution (BD Pharmingen, New Jersey, USA) after which sulphuric acid (2 M; 25 μl/well) was added to stop the colour reaction. The optical density (OD) of each well was measured at 450 nm using an LT-4000 Microplate Reader (Labtech, Uckfield, UK).

**Table 1:** Capture and detection antibodies screened for use in enzyme-linked immunosorbent assays for the detection of meerkat interferon-gamma (IFN-ɣ) and IFN-γ inducible protein 10 (IP-10)

Cytokine	Species	Manufacturer	Antibody catalogue no.	
			Capture	Detection
IFN-ɣ	Human	BD Pharmingen, New Jersey, USA	551221	554550
	Equine	Mabtech, Nacka Strand, Sweden	3117-1H-6	3117-1H-6
	Feline	R&D Systems Minnesota, USA	DY764	DY764
	Feline	AbD Serotech, Kidlington, UK	MCA2140	N/A
	Feline	Kingfisher Biotech, Minnesota, USA	PB0281F-100	PBB0283F-050
IP-10	Human	Peprotech, London, UK	900-K39	500-P93Bt
	Equine	Kingfisher biotech	PB0418E-100	PBB0423E-050
	Bovine	Kingfisher biotech	PB0385B-100	PBB0393B-050

### Antibody selection and ELISA optimisation

Selected anti-IFN-ɣ and anti-IP-10 antibodies (Table 1) were screened for potential reactivity to these cytokines in meerkat plasma as follows. Plasma from PWM-stimulated blood of 7 randomly selected animals was pooled and assayed using ELISAs comprising all possible combinations of selected capture and detection antibodies for either IFN-ɣ or IP-10. All antibodies were used at concentrations recommended by the manufacturer (Table 1). The antibody combination that resulted in the greatest OD values for each analyte was then selected and a dilution series of these antibodies was used in a checkerboard titration to assay pooled plasma samples from both PWM-stimulated and unstimulated blood. The optimal ELISA conditions were defined as the concentrations of capture and detection antibodies which resulted in the greatest relative OD difference derived from these samples.

### Cytokine release assay

Plasma samples, derived from whole blood which was processed as described above, were assayed in duplicate using the optimised ELISA. *M. suricattae*-specific cytokine release was defined as the OD obtained for the PC-HP-stimulated sample minus that for the sample co-incubated with PBS (OD^HP-Nil^). The PWM-stimulated plasma sample was used as a positive control for whole blood cytokine release and animals that showed greater cytokine release in whole blood co-incubated with PBS than in response to PWM stimulation (OD^PWM-nil^ < 0) were excluded from the study. Using the results from uninfected control animals, a diagnostic cut off value for this assay was calculated as the mean of all OD^HP-Nil^ values plus 2 standard deviations (SD).

### Statistical analysis

Using the Wilcoxon signed rank test, the release of IP-10 in blood co-incubated with PBS, PC-HP and PWM was compared for animals from the uninfected control group as well as those from the *M. suricattae*-exposed group. Data were analysed using GraphPad Prism 5 (GraphPad Software, Inc., La Jolla, USA). The performance of the cytokine release assay was evaluated in the free-living *M. suricattae-*exposed meerkat population by calculating the odds of an animal testing positive in each of the three infection risk categories defined above. These values were compared to that of category 1 using a chi-squared analysis.

## Results

### Development of a meerkat cytokine release assay

**Fig. 2:**
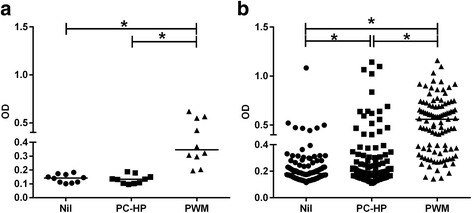
IP-10 release in whole blood incubated at 37 °C for 24 h with phosphate buffered saline (Nil), Bovigam PC-HP peptides and pokeweed mitogen (PWM), from **a** *Mycobacterium suricattae*-unexposed meerkats (*n* = 10), and **b** *M. suricattae*-exposed meerkats (n = 101). Significant differences between treatment conditions are indicated (Wilcoxon signed rank test, *p < 0.005)

Of the ELISAs evaluated for the detection of meerkat IFN-γ (Table 1), the assay comprising anti-human capture (2 μg/ml) and detection (2 μg/ml) antibodies resulted in the greatest OD value for PWM-stimulated samples, i.e. OD = 0.076. Of the IP-10 ELISAs (Table 1), the assay consisting of anti-bovine capture antibody (0.5 μg/ml) and anti-human detection antibody (0.25 μg/ml) resulted in the greatest ELISA signal, i.e. OD = 0.45. Hereafter, the IP-10 assay was selected for further analysis of PC-HP-stimulations and recombinant human IP-10 protein (Peprotech) was used as a positive control for the assay. Meerkats in the uninfected control group showed no significant difference in IP-10 release in blood incubated with PC-HP peptides and blood co-incubated with PBS (Fig. 2a). However, there was a significant difference in the release of this cytokine in PWM-stimulated blood and blood co-incubated with PBS (Fig. 2a). Using the IP-10 release assay (IPRA) test results (OD^HP-Nil^) from this group, a diagnostic cut off value was calculated as 0.038 (mean OD^HP-Nil^ + 2SD). This value was then used to classify meerkats from the low, intermediate and high infection risk categories as either IPRA-positive or -negative.

### Evaluation of test performance

A total of 108 *M. suricattae*-exposed meerkats were tested: 79 from social groups with no known history of TB (category 1), 11 from social groups with one or two deaths due to TB (category 2), and 18 from social groups with 3 to 7 deaths (category 3). For these animals, the OD^HP^ (median 0.20, interquartile range 0.16–0.32), and the OD^PWM^ (0.50, 0.32–0.69), were both significantly greater than the OD^Nil^ (0.19, 0.15–0.27) (Fig. 2b). The median OD^PWM-Nil^ value was 0.29 (range,−0.36 to 0.84) and 6/79, 0/11 and 1/18 animals from categories 1, 2 and 3, respectively, were excluded from further analysis on the basis of their OD^PWM-Nil^ result being less than zero. Of these 7 cases, 5 had OD^Nil^ values in the highest quartile of all samples.

For the remaining 101 meerkats, 35 animals had values greater than the diagnostic cut off (OD^HP-Nil^ > 0.038) (Table 2) with 24.7% testing positive in category 1, 27.3% in category 2 and 82.4% in category 3. The odds of an animal testing IPRA-positive was 14.0 times greater for animals with a high risk of *M. suricattae* infection (category 3) compared to animals with a low risk (category 1) (95% CI: 3.6–54.3, *p* < 0.0001).

**Table 2:** Analysis of IP-10 release assay (IPRA) results for 101 opportunistically sampled meerkats with a low, intermediate and high risk of infection with *M. suricattae*

Category	Infection risk (n)	Positive (%)	OR^a^ (95% confidence interval)	P
1	Low (73)	18 (24.7)	1	-
2	Medium (11)	3 (27.3)	1.13 (0.27–4.70)	0.87
3	High (17)	14 (82.4)	14 (3.61–54.34)	<0.0001
Total	101	35 (34.7)		

^a^OR, Odds ratio of an animal in a particular category testing positive, relative to category 1

## Discussion

We have developed a novel diagnostic assay for *M. suricattae* infection in meerkats which measures antigen-specific IP-10 release in whole blood incubated with Bovigam® PC-HP peptides. All meerkats with no known exposure to *M. suricattae* (*n* = 10) tested negative with the IPRA, while 35 out of 101 (34.6%) animals with known exposure to this pathogen tested positive. Although *M. suricattae* infection in these individuals was not confirmed, the odds of a meerkat testing positive were significantly greater for animals with a high risk of infection when compared to those with a low risk of infection, supporting the validity of the IPRA.

IP-10 has been shown to be a useful diagnostic biomarker for detection of *M. tuberculosis* infection in humans [9] and *M. bovis* infection in African buffaloes [11]. In both these species, IP-10 is produced in greater amounts than IFN-γ in antigen-stimulated whole blood and has been shown to be a more sensitive marker of mycobacterial infection [8, 9, 13]. In the present study, despite the use of ELISA antibodies produced against distantly related species, measurement of IP-10 proved to be a useful marker of immune activation. While this indicates the sensitivity of the ELISA for meerkat IP-10, it may, in part, reflect the abundance of this molecule in antigen-stimulated samples. Moreover, the similarity between the amino acid sequences of cattle, horses, cats and humans is significantly greater for IP-10 than for IFN-γ (data not shown) and this may explain the increased potential for cross-species reactivity of the anti-IP-10 antibodies. For these reasons, IP-10 may also be a useful diagnostic target in other species.

In order to define a diagnostic cut off value for the IPRA, we tested 10 captive meerkats with no known exposure to *M. suricattae*. These animals showed no significant difference in their IP-10 responses to PBS and PC-HP peptides indicating that their selection as uninfected controls was appropriate. Moreover, a threshold value of OD^HP-Nil^ > 0.038 classified all control animals as test-negative. Such a low threshold will increase the sensitivity of the assay, thereby reducing the number of false negative test results; however, this could be at the expense of specificity [14]. In part, this low cut off value resulted from plasma samples being diluted 1:4 in the present study and a lower dilution factor might improve test accuracy. However, this was not tested in the present study because of limited sample volumes.

In contrast to the control animals, 35 of 101 *M. suricattae*-exposed meerkats displayed significant IP-10 responses to the PC-HP peptides, confirming the antigenicity of this peptide pool for these animals. However, it is currently unclear which components of the PC-HP antigens might be responsible for this immune sensitization. The genetic region of difference 1 (RD1), a variant of which is deleted from the *M. suricattae* genome [15], encodes both ESAT-6 and CFP-10 and is also required for the secretion of Esx-1 substrate protein C (espC) which is encoded by the gene *Rv3615c* [16]. While *Rv*3615*c* is present in the *M. suricattae* genome (pers. comm., Anzaan Dippenaar), it is possible that this component of the PC-HP peptide pool, in addition to ESAT-6/CFP-10, would have a limited diagnostic contribution to detection of *M. suricattae*-infected animals. Nonetheless, the use of the PC-HP reagent is supported by the fact that the odds of an animal testing IPRA-positive were significantly greater for meerkats with the greatest risk of *M. suricattae* infection. Moreover, our results suggest that the PC-HP peptides might be useful for diagnostic testing of species infected with related RD1-deleted strains, i.e. *M. microti*, *M. mungi* and the dassie bacillus [5].

In both the captive and free-living populations, the median OD^PWM^ was significantly higher than the values for either OD^Nil^ or OD^HP^, indicating that PWM is an appropriate mitogen in this species. Seven animals were excluded from our analysis based on negative OD^PWM-Nil^ values; however, in 5 of these cases, the exclusion criterion was met as a result of unusually high OD^Nil^ values, not failure to respond to PWM. Similar spontaneous release of IP-10 has previously been seen in cattle [17], although the mechanism for this phenomenon is not currently understood. In the present study, although the positive control was intended to confirm the viability of cytokine production in blood samples, it additionally served to identify samples where high OD^Nil^ values would have affected the test interpretation. In such cases, repeat sampling of the animal would be recommended.

## Conclusion

The measurement of PC-HP induced IP-10 release is a useful biomarker of the risk of *M. suricattae* infection in meerkat populations. Moreover, while no *M. suricattae*-unexposed meerkats tested IPRA-positive, numerous animals from the exposed population with known cases of TB did, suggesting that the assay shows promise as a specific test for individual animals. Ongoing work which includes longitudinal testing and confirmation of infection status seeks to confirm the assay’s utility in individual meerkats. This IPRA will have use in further clarifying the epidemiology of *M. suricattae* infection in meerkats and test results may be used to inform management strategies for infected populations.

## Additional file

Additional file 1: IP-10 release in whole blood from *Mycobacterium suricattae*-unexposed meerkats (Control Group) and meerkats with a low risk (Category 1), moderate risk (Category 2) and high risk (Category 3) of infection with this pathogen. Blood was incubated at 37 °C for 24 h with phosphate buffered saline (Nil), Bovigam PC-HP peptides (HP) and pokeweed mitogen (PWM). The HP-specific (HP-Nil) and PWM-specific (PWM-Nil) IP-10 release was calculated by subtracting the Nil value from the HP and PWM values, respectively. (XLSX 21 kb)

## Abbreviations

BB: Blocking buffer
CFP-10: 10 kDa culture filtrate protein
CMI: Cell-mediated immunity
ELISA: Enzyme-linked immunosorbent assays
ESAT-6: 6 kDa early secretory antigenic target
espC: Esx-1 substrate protein C
IFN-ɣ: Interferon-gamma
IP-10: Interferon-gamma inducible protein 10
IPRA: IP-10 release assay
OD: Optical density
PBS: Phosphate buffered saline
PWM: Pokeweed mitogen
RD: Region of difference
SD: Standard deviation
TB: Tuberculosis
TMB: Tetramethylbenzidine
WB: Wash buffer
